# Assessment of Inner Retinal Layers and Choroidal Thickness in Type 1 Diabetes Mellitus: A Cross-Sectional Study

**DOI:** 10.3390/jcm8091412

**Published:** 2019-09-08

**Authors:** Marc Carbonell, Núria Alonso, Esmeralda Castelblanco, Jordi Real, Anna Ramírez-Morros, Rafael Simó, Cristina Hernández, Carme Jurjo, Alícia Traveset, Xavier Valldeperas, Dídac Mauricio

**Affiliations:** 1 Department of Ophthalmology, University Hospital Germans Trias i Pujol, 08916 Badalona, Spain; 2 Department of Surgery, Barcelona Autonomous University (UAB), 08035 Barcelona, Spain; 3 Department of Endocrinology and Nutrition, University Hospital and Health Science Research Institute Germans Trias i Pujol, 08916 Badalona, Spain (N.A.) (A.R.-M.); 4 Centre for Biomedical Research on Diabetes and Associated Metabolic Diseases (CIBERDEM), 08907 Barcelona, Spain (E.C.) (J.R.) (R.S.) (C.H.); 5 Department of Medicine, Barcelona Autonomous University (UAB), 08035 Barcelona, Spain; 6 Department of Endocrinology & Nutrition, Hospital de la Santa Creu i Sant Pau & Institut d’Investigació Biomèdica Sant Pau (IIB Sant Pau), 08041 Barcelona, Spain; 7 DAP-Cat Group, Unitat de Suport a la Recerca Barcelona, Fundació Institut Universitari per a la Recerca a l’Atenció Primària de Salut Jordi Gol i Gurina (IDIAPJGol), 08025 Barcelona, Spain; 8 Department of Endocrinology and Nutrition, Hospital Universitari Vall d’Hebron, Diabetes and Metabolism Research Unit, Vall d’Hebron Institut de Recerca (VHIR), Universitat Autònoma de Barcelona, 08035 Barcelona, Catalonia, Spain; 9 Department of Ophthalmology, University Hospital Arnau de Vilanova, 25198 Lleida, Spain (C.J.) (A.T.); 10 Biomedical Research Institute of Lleida (IRBLleida) & University of Lleida, 25198 Lleida, Spain

**Keywords:** type 1 diabetes, diabetic retinopathy, retinal nerve fibre layer, ganglion cell layer, choroid

## Abstract

Recent studies have shown that retinal neurodegeneration may precede visible vascular changes in diabetic retinopathy (DR). In addition, the relationship of choroidal thickness (CT) with DR stage is not well defined. To assess the inner retinal and choroidal structural changes in type 1 diabetic subjects (T1D), a cross-sectional study was conducted in 242 T1D patients and in 69 age-matched, non-diabetic individuals. The nasal retinal nerve fibre layer (RNFL) thickness was lower in T1D patients without DR (*p* < 0.001), with mild DR (*p* < 0.001), and with advanced DR (*p* < 0.001) compared to control subjects. The ganglion cell layer (GCL) thickness was lower in T1D patients with advanced DR compared to those with mild DR (*p* = 0.003) and without DR (*p* < 0.001) and compared to the control subjects (*p* < 0.001). T1D subjects with no DR and mild DR had higher CT than the control subjects, but the CT in T1D patients with advanced DR was lower (*p* = 0.038) than that in T1D subjects with mild DR and was not significantly different from that of the control subjects. In conclusion, T1D subjects showed a significant thinning of the nasal RNFL in the early stages of the disease, even before any vascular changes in the retina. A decrease in the GCL thickness during advanced DR stages was observed. Choroidal thickness was higher in T1D subjects without DR and in early DR stages but decreased in advanced stages.

## 1. Introduction

Diabetic retinopathy (DR) is one of the leading causes of visual impairment and preventable blindness among the adult working-age population in developed countries. DR is a multifactorial disease with many different risk factors, including glycated haemoglobin (HbA1c), blood pressure, and serum lipid levels [1,2]. Classically, DR has primarily been considered a microvascular disorder in which the initial manifestations include retinal microaneurysms, capillary alterations, and haemorrhaging. These vascular alterations may lead to degeneration of the neuronal and glial structures of the inner retina as the disease progresses. However, data from recent studies using spectral-domain optical coherence tomography (SD-OCT) imaging suggest that morphological changes in retinal neurodegeneration may precede any visible vascular changes [3,4,5,6,7]. In this regard, the earliest neurodegenerative alterations occur in the main layers of the inner retina, as evidenced by the thinning of the retinal nerve fibre layer (RNFL) and the ganglion cell complex (GCC) [4,6,7,8].

Choroidal alterations also play a key role in the pathophysiology of several retinal diseases, including DR [9,10,11,12]. Choroidal thickness (CT) is considered a measure of choroidal blood flow, which supplies the outer retinal layers. Several studies have described the presence of choroidal vasculopathy in diabetic subjects [9,13,14,15,16]. However, those studies—most of which were performed in a paediatric population—have reported contradictory findings, likely due to their small sample sizes. A similar controversy exists for the presence of retinal neurodegeneration in subjects who have diabetes without DR. The published data are contradictory, with some authors finding no differences in RNFL or ganglion cell layer (GCL) thickness in patients with type 1 diabetes (T1D) without DR compared to healthy control subjects [17,18], whereas other authors have described differences in these structures between diabetic patients and control subjects [19]. In this context, the aims of the present study were (1) to evaluate the retinal and choroidal structures in a large cohort of patients with T1D using SD-OCT and (2) to determine whether retinal and choroidal alterations are correlated with the degree of diabetic retinopathy.

## 2. Experimental Section

### 2.1. Patients

This was a cross-sectional study conducted in subjects diagnosed with T1D recruited from outpatient clinics at two university hospitals within the same health care organization (Institut Català de la Salut) in Catalonia, Spain.

The inclusion criteria for T1D subjects were as follows: (1) age ≥ 18 years, (2) diagnosis of T1D with disease duration ≥ one year, (3) normal renal function (estimated glomerular filtration rate > 60 mL/min), (4) urine albumin excretion rate < 300 mg/g, and (5) the absence of cardiovascular disease (ischaemic heart disease, cerebrovascular disease, peripheral arterial disease, and heart failure) or diabetic foot disease.

The control group consisted of 69 healthy age- and sex-matched volunteers. The selection criteria for the control subjects were the same as those for the T1D subjects, except for the specific microvascular complications (retinopathy and albuminuria). Additional inclusion criteria for the control subjects were: (1) fasting glucose < 100 mg/dL and (2) HbA1c < 5.7%. Ophthalmologic exclusion criteria for both groups included a refractive error of 5 dioptres or more, concomitant retinal diseases, ocular inflammation, glaucoma, macular oedema, previous surgery (during the year before the exam), and laser photocoagulation (during the six months before the exam).

The present subject sample was a subset of 340 T1D subjects included in a previously published study aimed at assessing the association of subclinical carotid atherosclerotic disease with the presence and severity of diabetic retinopathy [20]. For the current study, it was also important to exclude patients with previous cardiovascular disease and/or chronic kidney disease, as these two conditions are known to be associated with vascular damage. Of those 340 subjects, 98 were excluded from the present study either because they did not undergo SD-OCT (*n* = 78) or due to the presence of ophthalmologic exclusion criteria (*n* = 20) as follows: glaucoma (*n* = 3); laser treatments in the previous 6 months (*n* = 2); myopia > 5 dioptres (*n* = 6); and proliferative diabetic retinopathy treated with photocoagulation (*n* = 9). As a result, a total of 242 subjects were included in the current study.

### 2.2. Clinical Assessment

Serum and urine samples were collected in the fasting state. Subject characteristics (gender, age, and ethnicity), anthropometric parameters (weight, height, body mass index, and waist circumference), other parameters (hypertension, dyslipidaemia, smoking, medication, and disease duration) and laboratory measurements (glucose, HbA1c, triglycerides, total/HDL/LDL cholesterol, creatinine, and albumin/creatinine ratio), assessed using standard laboratory methods previously described, were recorded [21].

### 2.3. Ophthalmic Examination

A complete ophthalmological evaluation, including retinopathy classification, was performed by two expert ophthalmologists (MC and CJ) in both the patient and control groups. Optical coherence tomography measurements were performed using spectral-domain OCT (Cirrus HD-OCT, model 4000, Carl Zeiss Meditec, Dublin, CA, USA) to measure RNFL, GCL, and CT. Only SD-OCT scans with a signal strength > 8 were used for the analysis. RNFL thickness was measured according to an Optic Disc Cube 200 × 200 protocol, with the data calculated automatically by quadrant (temporal, superior, nasal, inferior). Mean values were also calculated. The GCL thickness was measured (including mean and minimum thicknesses) using the Macula Cube 512 × 128 protocol around the fovea. Choroidal thickness was measured using enhanced-depth imaging (EDI-OCT) with a high-definition 5-line Raster scan pattern. The measurements were made manually with a caliper in three positions (subfoveal, nasal, and temporal to the fovea) at a distance of 2000 µm. Choroidal thickness was calculated as the vertical distance between the first hyperreflective line (Bruch’s membrane) and the second hyperreflective line (the internal surface of the sclera) [22].

DR was classified into five stages according to the Early Treatment Diabetic Retinopathy Study (ETDRS) classification [23], as follows: (1) no apparent retinopathy, (2) mild non-proliferative retinopathy (NPDR), (3) moderate NPDR, (4) severe NPDR, and (5) proliferative diabetic retinopathy (PRD). Diabetic subjects with DR were reclassified into 2 stages (mild [ETDRS stages 1–2] or advanced [ETDRS stages 3–4] DR). Additionally, subjects with SD-OCT choroid measurements of 2 or more standard deviations above the mean value observed in the control group were classified as having a thickening of the choroid. Further, subjects with SD-OCT RNFL measurements less than 2 standard deviations below the mean value observed in the control group were classified as having a thinning of the RNFL. For the analysis, we used the ophthalmological variables obtained for the right eye if both eyes had the same DR grade. Otherwise, the variables from the eye with the highest DR grade were selected.

The ethics committees of both participating centres approved the study, which was performed in accordance with the principles of the Declaration of Helsinki. Written informed consent was obtained from all participants.

### 2.4. Statistical Analysis

A descriptive between-group comparison was performed to assess homogeneity in the clinical characteristics of the study population. Student’s *t*-test was performed to determine the significant differences between two groups (T1D versus control) in the quantitative variables, and a Chi-square test was used to compare qualitative variables between groups. Univariate and multivariate regression models were performed to estimate the crude and adjusted associations. The multivariate results were adjusted for sex, age, hypertension, dyslipidaemia, and body mass index (BMI).

The subanalysis in the T1D subjects was performed by examining the association between the DR grade and the ophthalmological variables. Differences between groups were evaluated by analysis of variance (ANOVA), and *p* values were corrected using the post hoc Tukey test. Linear multivariate regression models were performed to assess the associations for each ophthalmological variable. Correction for multiple comparisons was performed using the Benjamini–Hochberg procedure (or false discovery rate). The cut-off for statistical significance was set as *p* < 0.05. The statistical R package (v. 3.5.1, Free Software Foundation, https://www.r-project.org/) was used for data management and to perform the statistical analysis.

## 3. Results

The clinical characteristics of the study group are summarized in Table 1. Hypertension and dyslipidaemia were more prevalent in the T1D group, who also had higher blood pressure (systolic and diastolic) than the control subjects. There were no significant differences between groups in terms of age, sex, ethnicity, smoking habits, BMI, waist circumference, creatinine levels, or urine/albumin/creatinine ratios. The proportion of microalbuminuria was 3.3% (8/242) in T1D and 2.9% (2/69) in the control group. The mean duration of T1D was 20.6 years.

### 3.1. Ophthalmologic Examination

Of the 242 T1D subjects, 139 (57.4%) did not have DR, 74 (30.6%) had mild DR, and 29 (12%) presented with advanced DR. The clinical variables of the study group according to DR grade are shown in Table S1.

Nasal RNFL was significantly lower in all three T1D subgroups (i.e., those without DR, mild DR, and advanced DR) (*p* < 0.001) than in the control group, contrary to what was observed in the superior and inferior sectors, in which the RNFL was greater in the T1D subjects than in the control subjects *p* = 0.004 and *p* < 0.001, respectively. No significant differences between the T1D subjects and control subjects were observed in the temporal RNFL (*p* = 0.283) (Figure S1, Table S2). The proportion of subjects with retinal thinning assessed in the RNFL in T1D subjects without DR was 1.5% (2/130) in the superior sector, 0.8% (1/131) in the nasal sector, and 1.5% (2/131) in the inferior sector. The presence of retinal thinning in subjects with advanced DR was 11.1% (1/9) in the superior sector, 0% (0/9) in the nasal sector, and 22.2% (2/9) in the inferior sector.

**Figure 1:**
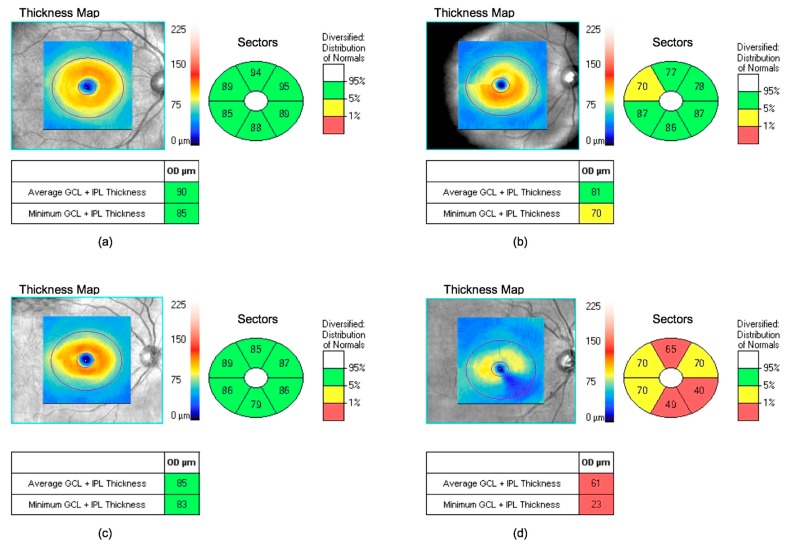
Ganglion cell layer (GCL) measurements by spectral-domain optical coherence tomography: (**a**) control subjects; (**b**) T1D subjects with mild DR; (**c**) type 1 diabetes (T1D) subjects without DR and (**d**) T1D subjects with advanced diabetic retinopathy (DR). Colours shown in the GCL thickness sector diagram should be interpreted as follows: white indicates expected values above 95%, green is between 5% and 95% (normal), yellow is from 1% to 5% (borderline), and red is below 1% (outside normal limits). OD, right eye.

GCL thickness was significantly lower in T1D subjects with advanced DR versus T1D subjects without DR (*p* < 0.001), subjects with mild DR (*p* = 0.010), and the control group (*p* < 0.001) (Table 2, Figure 1). There were no significant differences in GCL between the T1D and control subjects (Table S2).

**Figure 2:**
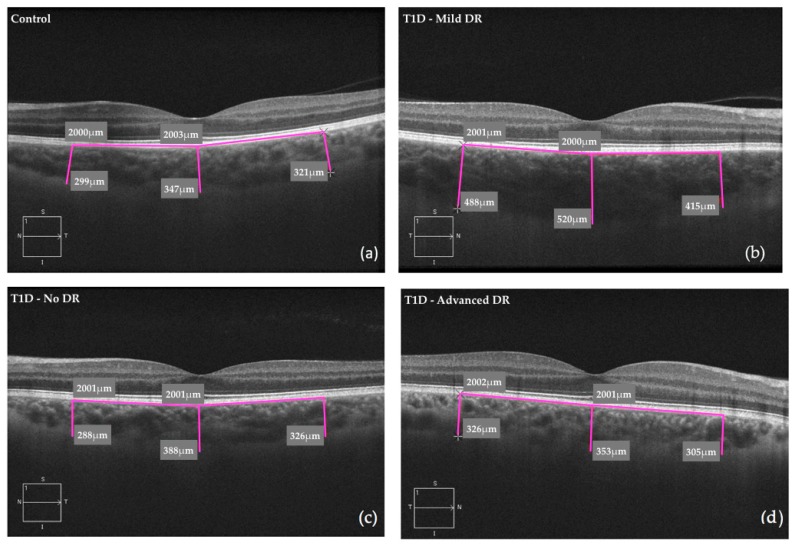
Choroidal measurements by spectral-domain optical coherence tomography: (**a**) control subjects; (**b**) T1D subjects with mild DR; (**c**) T1D subjects without DR and (**d**) T1D subjects with advanced DR. T1D, type 1 diabetes; DR, diabetic retinopathy.

**Figure 3:**
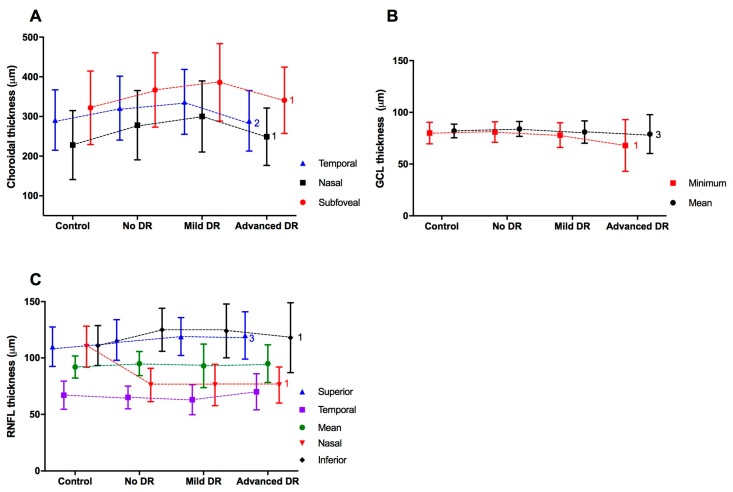
The thickness of the ophthalmological variables according to the diabetic retinopathy (DR) group. (**A**) Choroidal thickness. (**B**) Ganglion cell layer (GCL). (**C**) Retinal Nerve Fibre Layer thickness (RNFL). Differences between groups (control, no DR, Mild DR, and advanced DR) were evaluated by analysis of variance, and the *p* values were corrected using the post hoc Tukey test; 1, *p* < 0.001; 2, *p* < 0.01; 3, *p* < 0.05. In the figure, the mean and standard deviation. Control group *n* = 69, no DR *n* = 139, Mild DR *n* = 74 and Advanced DR *n* = 29.

Choroidal thickness was also significantly greater in the subfoveal (*p* < 0.001), nasal (*p* < 0.001), and temporal (*p* = 0.004) sectors in T1D subjects versus those in the control subjects (Figure 2). Up to 8.3% (11/133) of the T1D subjects without DR presented a thickening of the choroid. This proportion of patients was observed in the three sectors analysed (temporal, nasal, and subfoveal). In relation to the group of T1D subjects with mild DR, thickening was observed in 7.1% (5/70) of subjects in the temporal sector, 17.1% (12/70) in the nasal sector and 10% (7/70) in the subfoveal sector. Choroidal thickness was greater in the T1D subjects with mild DR and without DR compared to control subjects (Figure 2), both for the subfoveal region (*p* = 0.006 and *p* < 0.001, respectively) and the nasal region (*p* = 0.001 and *p* < 0.001, respectively). Temporal CT was greater only in patients with mild DR (*p* = 0.005) versus control subjects. Temporal and nasal CT values were lower in T1D subjects with advanced DR (*p* = 0.038 and *p* = 0.045, respectively) compared to T1D subjects with mild DR (Figure 3, Table 2).

### 3.2. Ophthalmological Associations

The univariate analysis revealed significant associations of T1D with subfoveal, nasal, and temporal CT and with RNFL thickness in the superior, nasal, and inferior sectors (Supplementary Table S3). Additionally, in T1D subjects without DR, thickening of the choroid was associated with age and smoking (*p* = 0.015 and *p* = 0.017, respectively) in the temporal sector and with smoking in the nasal sector (*p* = 0.004). Furthermore, in T1D subjects with mild DR, thickening of the choroid was associated with male gender in both the temporal sector (*p* = 0.017) and the subfoveal sector (*p* = 0.042) and with age and smoking habits in the subfoveal sector (*p* = 0.035 and *p* = 0.007, respectively).

In the multivariate linear regression analysis, all of these associations remained statistically significant after adjusting for clinical variables (sex, age, hypertension, dyslipidaemia, and BMI). Choroidal thickness was greater in T1D subjects compared to control subjects in all sectors: 50 µm in the subfoveal sector (β = 50.52; *p* < 0.001), 54 µm in the nasal sector (β = 54.99; *p* < 0.001), and 29 µm in the temporal sector (β = 29.08; *p* = 0.020). For RNFL thickness, the nasal sector was thinner in T1D patients compared to control subjects (β = −35.29, *p* < 0.001). However, RNFL values in the superior and inferior sectors were higher in T1D patients than in control subjects (β = 5.76, *p* = 0.046; β = 12.00, *p* < 0.001, respectively).

Multivariate regression analysis, performed to assess the association between the ophthalmological variables and the DR grade (mild and advanced DR versus no DR), showed that the minimum GCL was the only ophthalmological parameter independently associated with advanced DR. In this subject group, the minimum GCL value was 11 µm lower (β = −11.34; *p* = 0.004) than in T1D subjects without DR (Table 3). By contrast, no association was found between the DR grade and CT or RNFL values.

## 4. Discussion

In the present study, we found that, compared to control subjects, nasal RNFL thickness was lower in T1D subjects, regardless of the DR grade. GCL thickness was also lower in the advanced DR subgroup compared to subjects with mild DR or those without DR and to non-diabetic individuals. On the other hand, choroidal thickness was higher in patients with no DR and mild DR compared to the control subjects. Interestingly, CT was lower in subjects with advanced DR than in subjects with mild DR and similar to the CT values in control subjects. Thus, we report a direct correlation between choroidal alterations and the degree of retinopathy in T1D patients. Relatively few studies have evaluated the association between inner retinal and choroidal changes with the degree of DR in T1D patients [4,5,6,7,8,9,13,14,16,18,24]. Moreover, many of these studies were small, and most were performed in paediatric populations. Thus, the mean duration of diabetes was relatively short. To our knowledge, this study included the largest cohort of T1D patients with an evaluation of structural features of the retina by SD-OCT.

### 4.1. RNFL and GCL

There is evidence to suggest that retinal neurodegeneration begins early in the pathogenesis of DR [3,4,5,6,7,8,19,24,25]. In the early stages of DR, before this complication becomes clinically evident, it is not possible to detect microvascular abnormalities by ophthalmoscopic examination. However, in these pre-clinical stages, the presence of abnormalities can be detected by multifocal electroretinogram (mfERG) or SD-OCT [26]. Some authors have found no differences in the RNFL or GCL thickness using SD-OCT in paediatric [17,18] or young [9] T1D subjects without DR when compared to non-diabetic subjects [18]. By contrast, other studies have found differences in RNFL and GCL thickness when comparing diabetic subjects to control subjects. A meta-analysis that included 8 studies with small sample sizes concluded that retinal thinning may be present in diabetic subjects with mild DR or no DR, leading those authors to suggest that retinal neurodegeneration may be considered an early stage of DR that precedes visible retinal vasculopathy [19]. Other studies have compared young or paediatric T1D subjects without DR to healthy control subjects and found significant thinning of the RNFL [4,5,8] and the GCL [4,5,7,8] in diabetic patients. Gundogan et al. found that GCL thinning was most prominent in the temporal sector, leading those authors to suggest that the temporal GCL sectors are the most vulnerable in diabetic subjects and, thus, that data obtained by SD-OCT could be used as biomarkers of retinal neurodegeneration in diabetic patients without DR [5]. We did observe a significant thinning in the GCL of T1D subjects compared to the control subjects, although this change was only significant for advanced DR, not for mild or no DR. However, we were unable to evaluate these sectorial changes in the temporal GCL area because the SD-OCT instrument used does not allow for this type of subanalysis. Van Dijk et al. found RNFL and GCL thinning in T1D subjects with mild DR but not without DR compared to control subjects [6]. Their results suggest that thinning of the inner retinal layers appears to occur at the expense of thinning of the GCL and later of the RNFL due to loss of ganglion cell axons. These authors inferred that the nasal RNFL sector is the most vulnerable in diabetic subjects, since ganglion cell axons travel in bundles to the head of the optic nerve without crossing. We found similar changes in our series, with an intense thinning of the nasal RNFL in T1D subjects without DR compared to control subjects as reported by other authors [8]. Notably, this thinning was similar in subjects with advanced stages of DR. Importantly, this finding remained statistically significant after adjustment for clinical variables in the multivariate regression analysis. It is interesting to note that the presence of any sign of macular oedema was a strict exclusion criterion, and, therefore, macular thickness (or the thickness of any of the inner retinal layer) was not affected by this feature in our patients. Other authors obtained similar results in studies based on mfERG, in which they found a longer P1-implicit time and a lower P1 amplitude in the nasal area than in the temporal area in eyes of diabetic subjects [27,28].

More recently, the results reported in the European Consortium for the Early Treatment of Diabetic Retinopathy (EUROCONDOR) study, which enrolled 449 participants with type 2 diabetes, pointed to the existence of two phenotypes of subjects: a group in which there is neurodegeneration/neurodysfunction without microvascular involvement and another group of subjects with microangiopathy but without neurodysfunction. The results of the study showed that 58% of subjects with neurodysfunction, evaluated by mfERG, had no evidence of visible retinopathy. On the other hand, 32% of subjects with visible microvascular disease did not present any functional or structural abnormalities related to neurodegeneration. This study also showed that the correspondence between SD-OCT thinning and mfERG abnormalities in subjects without DR was 67% [26,29]. Our results are in line with those reported in the EUROCONDOR study, in that there is a proportion of subjects without DR who show SD-OCT evidence of neurodegeneration, while a proportion of subjects with severe nonproliferative DR do not show SD-OCT evidence of neurodegeneration.

### 4.2. Choroid

The choroid provides oxygen and nutrients to the outer retinal layers and is the only blood supply of the avascular fovea [30]. In DR, several histopathological choroidal abnormalities have been reported, including vascular remodelling with increased tortuosity, obstruction and dilatation of the choriocapillaries, choroidal aneurysms, and choroidal neovascularisation. The association between choroidal thickness and DR stage has been widely reported. Several studies have not found any significant differences between T1D subjects and control subjects in terms of CT [8,9,16,24]. However, this lack of difference could be due to the relatively recent onset of diabetes in the patients (mostly paediatric) included in those studies. By contrast, other studies have found significant choroidal thinning in T1D patients with [14] and without DR [5,14] compared to non-diabetic individuals. Vusojevic et al. attributed this thinning to a relative vasoconstriction or decrease in perfusion pressure in the choroidal vessels [15]. Our results—obtained in a large cohort of adult T1D subjects—differ substantially from those of previous reports. We found a significant increase in CT in diabetic subjects with mild DR and even in those without DR. Interestingly, while choroidal thickening was observed in diabetic subjects without DR and in those with mild DR, the CT in patients with advanced DR was similar to the CT in controls. A possible explanation for the CT difference observed in relation to retinopathy grades may be related to inflammation. It has been suggested that polymorphonuclear neutrophils might contribute to vaso-occlusive events and endothelial cell injury in the diabetic choroid. In this way, the loss of choriocapillaries in the diabetic choroid could render those areas of the choroid overlaying the retinal pigment epithelium (RPE) hypoxic [31]. When the RPE is hypoxic, it upregulates VEGF (vascular endothelial growth factor) production, which stimulates angiogenesis [32]. Alternatively, the increase in CT could be interpreted as a hyper-vascularization stage similar to the hyperfiltration that occurs in early stages of diabetic nephropathy. These findings suggest that changes in choroidal vasculature could be an early event in the diabetic retina, even where there is no DR [33]. The thinner CT observed in the advanced stages of DR could be related to the progressive damage of the endothelium that results from this process, causing reduced flow and ischaemia.

### 4.3. Study Strengths and Limitations

The present study has several limitations. First, despite the relatively large number of subjects with T1D included in the study, after stratification by the degree of DR, the numbers in each category were limited, especially in the advanced stages. Second, patients with a history of cardiovascular disease, microalbuminuria, or diabetic foot disease were excluded from the study, thereby excluding patients with other comorbidities. Third, the presence of retinal neurodegeneration was assessed by SD-OCT to measure the inner retinal layer thickness (a morphological parameter), whereas the use of other technologies based on the assessment of functional parameters (e.g., mfERG or microperimetry) might exhibit different results. Fourth, the refractive error of the patients included in the study was not recorded. Since myopic status could alter chorioretinal thickness, especially in high myopia, the absence of data on refractive errors can also be added to the limiting factors. However, it should be noted that all patients had less than 5.00 dioptres. Finally, the absence of patients without previous established cardiovascular and/or renal disease does not enable us to extrapolate our findings to T1D subjects with previous cardiovascular disease and/or chronic kidney disease.

This study also has several important strengths. First, this study included the largest cohort of patients with T1D, in which SD-OCT was used to evaluate structural features of the retina. In addition, it reports, for the first time, a direct correlation between choroidal alterations and the degree of diabetic retinopathy in patients with T1D.

## 5. Conclusions

The current study, carried out on T1D patients, reveals a significant thinning of nasal RNFL, even before any vascular changes in the retina can be detected by standard diagnostic techniques. GCL thickness was also decreased in T1D subjects with advanced DR compared to the control subjects. In addition, while choroidal thickness was increased in T1D subjects with mild or no DR, the CT thickness in subjects with advanced DR was similar to the CT values obtained from the control subjects. Overall, our findings suggest that SD-OCT could be used as an early diagnostic imaging test to detect diabetes-related retinal involvement.

## Figures and Tables

**Table 1:** Clinical characteristics of the study population.

Variables ^1^	Control	Type 1 Diabetes	*p* Value
*N*	69	242	-
Age, years	45.1 (11.2)	44.5 (10.7)	0.668
Sex, male	30 (43.5%)	114 (47.1%)	0.692
Ethnicity, Caucasian	69 (100%)	239 (98.8%)	1.000
Current or former smoker	35 (50.7%)	128 (53.2%)	0.387
Antiplatelet agents	0 (0.00%)	67 (27.7%)	<0.001
Dyslipidaemia	5 (7.25%)	95 (39.3%)	<0.001
Hypertension	7 (10.1%)	58 (24.0%)	0.020
Systolic blood pressure, mmHg	118 (13.2)	126 (17.1)	<0.001
Diastolic blood pressure mmHg	71.8 (9.38)	74.4 (10.0)	0.049
Body mass index, kg/m^2^	24.6 (3.62)	25.6 (4.05)	0.059
Waist circumference, cm	87.2 (11.0)	88.9 (12.6)	0.290
HbA1c, %	5.28 (0.31)	7.60 (1.03)	<0.001
Total cholesterol, mg/dL	203 (30.8)	180 (28.9)	<0.001
HDL cholesterol, mg/dL	59.9 (12.4)	64.3 (15.4)	0.017
LDL cholesterol, mg/dL	123 (29.3)	102 (24.2)	<0.001
Triglycerides, mg/dL	103 (69.4)	74.4 (34.7)	0.002
Creatinine, mg/dL	0.79 (0.13)	0.77 (0.16)	0.151
Urine albumin/creatinine ratio, mg/g	7.65 (10.4)	7.02 (13.6)	0.684
Diabetes duration, years	-	20.6 (10.4)	-

^1^ All data are given as the mean (standard deviation) or *n* (%). Student’s *t*-test was used for quantitative variables and Chi-square test for qualitative variables. HDL, high density lipoprotein; LDL, low density lipoprotein.

**Table 2:** Association between diabetic retinopathy grade and ophthalmological variables.

Variables ^1^	Control	No DR	Mild DR	Advanced DR	*p* Overall	*p* CT vs. No DR	*p* CT vs. Mild DR	*p* CT vs. Adv. DR	*p* No DR vs. Mild DR	*p* No DR vs. Adv. DR	*p* Mild DR vs. Adv. DR
CT (subfoveal)	322 (92.6)	367 (93.8)	386 (97.7)	341 (83.7)	<0.001	0.006	<0.001	0.812	0.549	0.526	0.148
CT (nasal)	228 (86.9)	278 (87.4)	300 (89.5)	249 (72.4)	<0.001	0.001	<0.001	0.706	0.314	0.372	0.045
CT (temporal)	291 (76.2)	321 (80.5)	337 (81.8)	289 (76.3)	0.002	0.056	0.005	0.998	0.542	0.206	0.038
GCL (mean)	82.4 (6.6)	84.3 (7.2)	81.4 (10.8)	78.5 (18.8)	0.018	0.561	0.925	0.275	0.175	0.024	0.533
GCL (minimum)	79.6 (10.4)	80.7 (9.9)	77.8 (12)	68 (25.0)	<0.001	0.932	0.821	<0.001	0.373	<0.001	0.003
RNFL (mean)	91.6 (9.8)	95.1 (10.7)	92.5 (19.3)	94.6 (16.7)	0.287	0.305	0.980	0.763	0.545	0.998	0.898
RNFL (temporal)	66.9 (12.5)	65.4 (10.1)	62.9 (13.3)	70.2 (16)	0.056	0.822	0.197	0.681	0.499	0.303	0.063
RNFL (superior)	110 (17.5)	116 (18.0)	119 (16.8)	120 (21)	0.026	0.104	0.035	0.141	0.837	0.852	0.995
RNFL (nasal)	110 (18.2)	75.9 (14.8)	75.5 (18.3)	76.4 (16.0)	<0.001	<0.001	<0.001	<0.001	0.999	0.999	0.997
RNFL (inferior)	111 (17.7)	125 (19.1)	124 (23.9)	118 (31.0)	<0.001	<0.001	0.003	0.484	0.954	0.471	0.725

^1^ All data are given as the mean (standard deviation), measure units in µm. Differences between groups were evaluated by analysis of variance (ANOVA), and *p* values were corrected using the post hoc Tukey test. Adv., Advanced; CT, choroidal thickness; GCL, ganglion cell layer; RNFL, retinal nerve fibre layer.

**Table 3:** Multivariate analysis of the association between the ophthalmological variables and the grade of diabetic retinopathy in patients with type 1 diabetes.

Variables	Mild DR		Advanced DR	
	Estimated β (SE)	*p* Value	Estimated β (SE)	*p* Value
CT (subfoveal)	28.17 (13.31)	0.126	−7.52 (20.50)	0.892
CT (nasal)	28.84 (12.64)	0.126	−13.75 (19.46)	0.687
CT (temporal)	23.83 (11.40)	0.126	−17.46 (17.56)	0.535
GCL (mean)	−2.39 (1.52)	0.330	−4.96 (2.36)	0.126
GCL (minimum)	−2.06 (1.91)	0.514	−11.34 (2.98)	0.004
RNFL, mean	−2.43 (2.12)	0.514	0.54 (3.34)	0.917
RNFL, temporal	−2.48 (1.78)	0.412	6.99 (3.01)	0.126
RNFL, superior	2.23 (2.69)	0.629	1.17 (4.56)	0.917
RNFL, nasal	−0.22 (2.43)	0.929	0.71 (4.11)	0.917
RNFL, inferior	−1.34 (3.28)	0.892	−6.10 (5.56)	0.514

In the multivariate analysis, the estimated β coefficients are given after adjustment for the clinical variables (sex, age, hypertension, dyslipidaemia, and body mass index), and *p* values were adjusted via the method of Benjamini and Hochberg for multiple comparisons. SE, standard error; CT, Choroidal thickness; GCL, ganglion cell layer; RNFL, retinal nerve fibre layer.

## Supplementary Materials

The following material is available online at https://www.mdpi.com/2077-0383/8/9/1412/s1: Table S1: Clinical characteristics of the study population according to diabetic retinopathy grade; Table S2: Ophthalmological variables in the study population by group; Table S3: Univariate and multivariate analysis of the association between ophthalmological variables and type 1 diabetes with respect to the control group; and Figure S1: Retinal nerve fibre layer (RNFL) measurements by spectral-domain optical coherence tomography: (a) control subjects and (b) T1D subjects with mild DR. TEMP, temporal sector; SUP, superior sector; NAS, nasal sector; INF, inferior sector.
