# Supplementary material for: Assessment of Inner Retinal Layers and Choroidal Thickness in Type 1 Diabetes Mellitus: A Cross-Sectional Study
Source: J Clin Med. 2019 Sep 8;8(9):1412. doi: 10.3390/jcm8091412 (PMC6780763; doi:10.3390/jcm8091412)
Supplement: Supplementary file 1 [file jcm-08-01412-s001.pdf]

Journal of
Clinical Medicine

MDPI

Table S1. Clinical characteristics of the study population according to diabetic retinopathy grade.

Variables[1]	Control N=69	No DR N=139	Mild DR N=74	Advanced DR N=29	*p* overall	*p* CT vs No DR	*p* CT vs Mild DR	*p* CT vs Adv. DR	*p* No DR vs Mild DR	*p* No DR vs Adv. DR	*p* Mild DR vs Adv. DR
Age, years	45.1 (11.2)	42.8 (10.1)	45.9 (11.5)	49.0 (9.78)	0.018	0.440	0.972	0.358	0.176	0.023	0.548
Sex, men	30 (43.5%)	68 (48.9%)	33 (44.6%)	13 (44.8%)	0.869	1.000	1.000	1.000	1.000	1.000	1.000
Race, Caucasian	69 (100%)	137 (98.6%)	73 (98.6%)	29 (100%)	1.000	1.000	1.000	.	1.000	1.000	1.000
Current/former smoker	35 (50.7%)	72 (52.2%)	40 (54.1%)	15 (55.2%)	0.901	0.981	0.981	0.981	0.981	0.981	0.981
Antiplatelet Agents	0 (0.00%)	36 (25.9%)	18 (24.3%)	13 (44.8%)	<0.001	<0.001	<0.001	<0.001	0.931	0.086	0.086
Dyslipidaemia	5 (7.25%)	48 (34.5%)	32 (43.2%)	15 (51.7%)	<0.001	<0.001	<0.001	<0.001	0.325	0.190	0.577
Hypertension	7 (10.1%)	19 (13.7%)	20 (27.0%)	19 (65.5%)	<0.001	0.616	0.027	<0.001	0.032	<0.001	0.001
Systolic BP, mmHg	118 (13.2)	123 (15.9)	130 (15.5)	135 (22.3)	<0.001	0.203	<0.001	<0.001	0.019	0.003	0.523
Diastolic BP mmHg	71.8 (9.38)	74.7 (10.3)	74.3 (8.48)	73.2 (12.5)	0.249	0.202	0.443	0.912	0.992	0.903	0.968
Body mass index, kg/m^2	24.6 (3.62)	25.2 (3.99)	25.9 (3.73)	26.6 (4.97)	0.095	0.709	0.242	0.118	0.699	0.355	0.845
Waist circumference, cm	87.2 (11.0)	88.0 (11.8)	89.7 (11.7)	91.1 (17.4)	0.426	0.972	0.653	0.496	0.810	0.627	0.955
HbA1c, %	5.28 (0.31)	7.38 (0.88)	7.78 (1.15)	8.18 (1.09)	<0.001	<0.001	<0.001	<0.001	0.011	<0.001	0.181
HbA1c, mmol/mol	34.2 (3.57)	57.2 (9.56)	61.6 (12.6)	65.8 (11.9)	<0.001	<0.001	<0.001	<0.001	0.011	<0.001	0.190
Total cholesterol, mg/dL	203 (30.8)	179 (26.2)	181 (31.8)	186 (33.4)	<0.001	<0.001	<0.001	0.045	0.916	0.578	0.871
HDL, mg/dL	59.9 (12.4)	64.4 (14.4)	63.8 (17.5)	65.0 (15.3)	0.191	0.174	0.417	0.406	0.991	0.997	0.979
LDL, mg/dL	123 (29.3)	101 (21.7)	102 (25.9)	106 (30.5)	<0.001	<0.001	<0.001	0.019	0.970	0.685	0.883
Triglycerides, mg/dL	103 (69.4)	71.6 (29.3)	79.5 (43.8)	75.1 (31.9)	<0.001	<0.001	0.011	0.028	0.611	0.981	0.970
Creatinine, mg/dL	0.79 (0.13)	0.77 (0.17)	0.75 (0.14)	0.79 (0.16)	0.285	0.736	0.289	1.000	0.742	0.866	0.508
Albumin/creatinine ratio, mg/g	7.65 (10.4)	4.99 (4.76)	5.30 (7.37)	21.2 (33.4)	<0.001	0.456	0.661	<0.001	0.998	<0.001	<0.001
Diabetes duration, years	-	17.0 (9.00)	24.8 (10.4)	27.6 (8.94)	<0.001	-	-	-	<0.001	<0.001	0.354

[1]All data are given as the mean (standard deviation) or n (%). BP, blood pressure; HDL, high density lipoprotein; LDL, HDL, low density lipoprotein.

Journal of
Clinical Medicine
MDPI

Table S2. Ophthalmological variables in the study population by group.

Variables[1]	Control	T1D	*p* value
Subfoveal CT	322 (92.6)	370 (94.5)	<0.001
Nasal CT	228 (86.9)	281 (87.5)	<0.001
Temporal CT	291 (76.2)	322 (81.3)	0.004
GCL Average	82.4 (6.64)	82.8 (10.4)	0.764
GCL Minimum	79.6 (10.4)	78.4 (13.6)	0.417
RNFL (Average)	91.6 (9.81)	94.2 (14.6)	0.082
RNFL (Temporal)	66.9 (12.5)	65.1 (12.0)	0.283
RNFL (Superior)	110 (17.5)	117 (17.9)	0.004
RNFL (Nasal)	110 (18.2)	75.8 (16.0)	<0.001
RNFL (Inferior)	111 (17.7)	124 (22.0)	<0.001

[1]All data are given as the mean (standard deviation), measure units in μm and *p* values were adjusted by the method of Benjamini & Hochberg for multiple comparisons. RNFL, retinal nerve fibre layer; CT, choroidal thickness; GCL, ganglion cell layer.

Table S3. Univariate and multivariate analysis of the association between ophthalmological variables and type 1 diabetes with respect to the control group**.**

Variables	Univariate		Multivariate	
	Estimated β (SE)	*p* value	Estimated β (SE)	*p* value
Subfoveal CT	48.03 (12.90)	**<0.001**	50.52 (13.28)	**<0.001**
Nasal CT	53.75 (11.98)	**<0.001**	54.99 (12.57)	**<0.001**
Temporal CT	30.80 (10.99)	**0.009**	29.08 (11.24)	**0.020**
GCL, mean	0.32 (1.33)	0.813	1.09 (1.39)	0.482
GCL, minimum	-1.24 (1.77)	0.537	-0.55 (1.84)	0.776
RNFL (mean)	2.65 (1.87)	0.226	1.62 (1.97)	0.482
RNFL (temporal)	-1.84 (1.67)	0.338	-1.82 (1.77)	0.436
RNFL (superior)	7.08 (2.46)	0.009	5.76 (2.60)	0.046
RNFL (nasal)	-33.97 (2.28)	<0.001	-35.29 (2.43)	<0.001
RNFL (inferior)	13.20 (2.91)	<0.001	12.00 (3.07)	<0.001

In the multivariate analysis, estimated β coefficients are given after adjusting for the clinical variables (sex, age, hypertension, and dyslipidaemia and body mass index), and *p* values were adjusted by the Benjamini & Hochberg procedure for multiple comparisons. The control group was used as a reference group. SE, standard error; CT, Choroidal thickness; GCL, ganglion cell layer; RNFL, retinal nerve fibre layer.

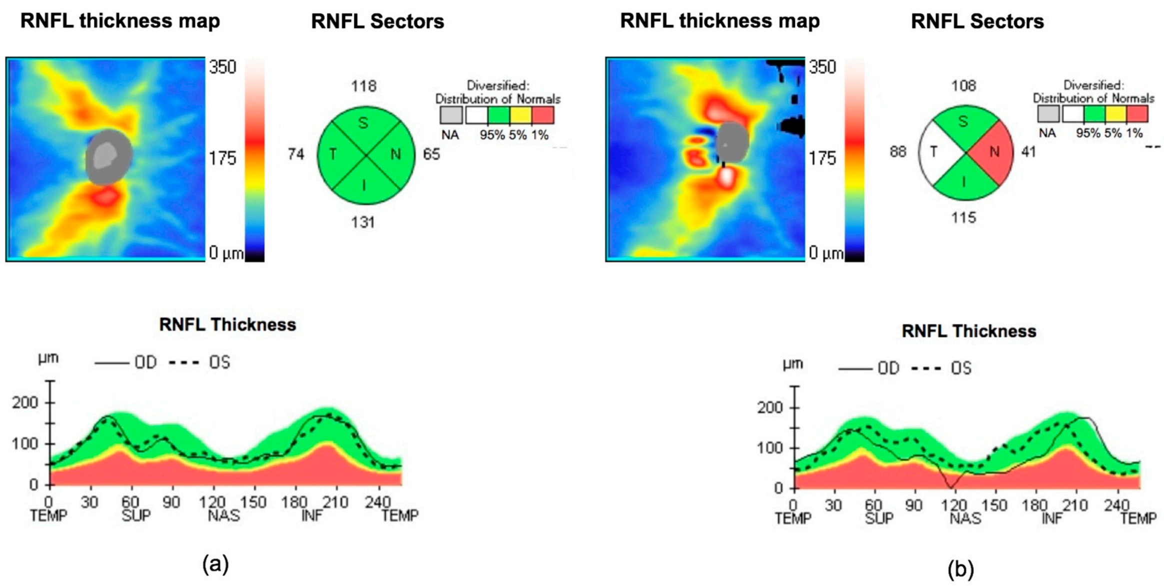

Figure S1. Retinal nerve fibre layer (RNFL) measurements by spectral-domain optical coherence tomography: (a) control subjects and (b) T1D subjects with mild DR. TEMP and T, temporal sector; SUP and S, superior sector; NAS and N, nasal sector; INF and I, inferior sector. Colours shown in the RNFL thickness sector diagram should be interpreted as follows: white indicates expected values above 95%, green between 5 and 95% (normal), yellow from 1 to 5% (borderline), and red below 1% (outside normal limits). OD, right eye; OS, left eye.
